# Rituals of Containment: Many Pandemics, Body Politics, and Social Dramas During COVID-19 in Pakistan

**DOI:** 10.3389/fsoc.2021.648149

**Published:** 2021-04-30

**Authors:** Inayat Ali

**Affiliations:** Department of Social and Cultural Anthropology, University of Vienna, Vienna, Austria

**Keywords:** COVID-19, death rituals, many pandemics, political pandemic, social pandemic, body politics, social drama, Pakistan

## Abstract

Infecting millions of people, causing around two million deaths, and affecting billions of people worldwide during January 2021, the coronavirus 2019 (COVID-19) pandemic is not merely one pandemic but many. These many pandemics, which I identify herein, have revealed the overt and subtle entanglements among religion, science, and politics around COVID-19. Building on my current ethnographic research on COVID-19 using purposive sampling and interview guide in Pakistan, and borrowing from various anthropological concepts such as “social drama,” proposed by Victor Turner, and ritual, I have developed a concept that I call *rituals of containment*. With this concept, I extend my previous argument regarding “symbolic ownership” to show a visible “body politics” by demonstrating how religion, science, and politics around COVID-19 are entangled at individual and government levels. This has become observable through the rituals of the Pakistani government of containment to deal with COVID-19. Such entanglements are visible in the case of strategies to tackle infected “viral bodies,” as the government has enacted its authority: (1) to bury what I am terming the *dead viral body* without its beloved ones present; (2) to return or not to return this body to family members in a coffin; (3) or to provide the grieving family with a symbolic empty coffin. These Covidian politics have led to the question: Who in actuality owns the body? In conclusion, I argue that the problem lies in the discriminatory and contradictory rituals of containment of the government, not in using scientific evidence and guidelines.

## Introduction: Multiple Pandemics

Starting in late 2019 and becoming a pandemic in the spring of 2020, the almost yearlong the coronavirus 2019 (COVID-19) pandemic has directly or indirectly, visibly or invisibly affected us all. By January 2021, the novel coronavirus had infected around 102 million people and caused around 2.2 million deaths worldwide (Johns Hopkins University, 2021). These epidemiological data, although significant, demonstrate only some of the critical implications of the pandemic. Numbers alone cannot reveal the structured vulnerabilities and politics at play around COVID-19, yet critical anthropological analysis can. From an anthropological perspective, I see COVID-19 as much more than a medical pandemic. It is also an “economic pandemic,” a “social pandemic,” a “structural pandemic,” an “emotional/psychological pandemic,” and a “political pandemic.”

With the term “economic pandemic,” I index the financial or resource-related impacts of the pandemic as, globally, COVID-19 has caused and is continuing to cause a significant increase in poverty (see Pereira and Oliveira, 2020). By “social pandemic,” I mean the implications of COVID-19 for social systems worldwide, as values, norms, and mores have been greatly influenced and significantly changed, including those within individual families. With “structural pandemic,” I refer to the social, ethnic, geographic, and economic stratifications that have allowed the impacts of this disease to fall most heavily on the poor and marginalized.

The term “emotional/psychological pandemic” connotes the enormous emotional and psychological tolls the coronavirus pandemic has taken on individuals around the world (see for example Cullen et al., 2020). These include the emotional impacts on families due to the interruption of death rites caused by the common prohibitions on group gatherings, often robbing people of the chance to say their final goodbyes (Gonçalves Júnior et al., 2020). I use the label “political pandemic” to index the contested politics and governmental mistrust that have been woven around COVID-19 at local, national, and global levels (see Liu and Bennett, 2020). The well-publicized political contestations between China and the US constitute a good example (Ali, 2020b,c), as does the mistrust shown by many Pakistanis toward the government viral containment policies, which they perceive as efforts to monitor them and control their movements (Ali, n.d.). Different narratives have emerged worldwide (Ali, 2020b,c; Mukhtar, 2021) as politicians have played various “political cards,” such as using militarized terminology and invoking religion, patriotism, or nationalism in the “war” against the coronavirus (see Whyte, 2020). These metaphors are “conceptual apparatuses,” or “rhetorical tool[s]” embedded in sociocultural, political, economic, and ideological structures and processes that influence imaginaries and behaviors (Bates, 2020; Castro Seixas, 2021). Revealing “securitization” (Buzan et al., 1998), metaphors have exposed “the discursive acts of justification of extraordinary means to eliminate a threat” while working “as a strategic persuasive master frame that is articulated by elites and passed through media coverage, to convince various publics of the appropriateness of the employed [containment] measures” (Lukacovic, 2020).

All of these pandemics, be it economic, social, structural, emotional/psychological, or political, are relevant to my discussion below of *dead viral bodies* and how these are treated by the Pakistani government, as they all involve, what I term, *rituals of containment*. Informed by the definition by Robbie Davis-Floyd (2003 [1992]) of a ritual as “a patterned, repetitive, and symbolic enactment of a cultural (or individual) belief or value,” by this term, I index dealings of the Pakistani government (and, later on, of the individual families) with the pandemic. Such rituals symbolically enact the values of the government on the kinds of politics that employ religion, language, and science to accomplish its goals. In these rituals of containment, which involve containing the virus, containing the bodies it kills, and containing the emotional reactions of people to its treatment of these dead viral bodies, the Pakistani government, like many worldwide, exerts what Galtung (1969) called “invisible violence” or what Paul Farmer (2004) termed “structural violence” or Kleinman et al. (1997) called “social suffering.”

Building on my previous work on shifting the meaning of a “normal body” to a “viral body” in Pakistan, which is a concept I developed to index the still-contagious nature of a body that died due to COVID-19 (Ali, 2021), and on the relevant literature, herein, I will construct a thorough picture of rituals of containment.

## Methods and Materials

### Research Design

It is indispensable to mention that this is a brief report article that is part of my long-term project on COVID-19, approved by Pakistan's National Bioethical Committee (reference No. 4-87/NBC-471-COVID-19-09/20/). Building on various data resources, primarily ethnographic fieldwork conducted during the COVID-19 pandemic initially reported in Pakistan in March 2020, I have adopted a qualitative research study design with an interview guide to gathering data, so as to study and comprehend the perspectives on COVID-19 in Pakistan. The central question of the entire COVID-19 project is how various stakeholders such as laypeople and the government have dealt with the pandemic in Pakistan and how they have negotiated and contested it. Since this is an ongoing project to explore the impact of the pandemic, including on death rites and mourning rituals, I draw on interviews of a few interlocutors who experienced the deaths of their dear ones due to COVID-19. All the interlocutors were informed about the project and asked to give their consent.

### Data Collection and Analysis

Using the purposive sampling method to select interlocutors and employing an interview guide, I started collecting the data virtually and physically with the help of a small team in Pakistan. Under this project, around 300 individual/narrative interviews, informal discussions, *Kachahari* sessions, and two online surveys were conducted from March 2020 to April 2020 and January 2021. Kachahari is similar to a group discussion but has significant dissimilarities as it is a socio-culturally rooted qualitative method that allows interlocutors to lead the discussion and be at ease, as opposed to a focus group discussion in which the researcher leads (Ali, 2020a). Also, I did content and document analysis of news reports and various surveys, mainly government reports, to contextualize the pandemic in Pakistan. The data were collected from Sindh and Punjab provinces in the Sindhi, Seraiki, and Urdu languages and later were transcribed into English and thematically examined. In addition to these current data, I also build on my almost 15 years of long-term ethnographic fieldwork conducted for my Master's, M.Phil., and Ph.D. degrees in the country, including Sindh province, with a focus on studying and analyzing various forms of politics and disparities, especially those revolving around infectious diseases and vaccination programs.

As far as the data analysis is concerned, it commenced right from the beginning of the data collection in March 2020. The data gathered from interviews and media were subjected to content analysis. To be familiarized with the data and allow for iteration, I constantly read and re-read it and have identified salient themes.

## Rituals of Containment: Social Drama, Metaphors, and Securitization

When one panoramically revisits the effects of the pandemic and various strategies to deal with it, one can observe a sequence of repetitive activities involving policies, practices, gestures, words, and actions (see Leach, 1966; Bloch, 1974; Davis-Floyd, 2003 [1992]) that have been performed at local, national, and global levels. Again, I call such repetitive performances “rituals of containment,” as in, rituals created to contain the viral spread in various ways and for other, related purposes. Since such rituals of containment have tended to construct and perpetuate forms of social class, authority, and power, these performances can also be viewed as political rituals (see Geertz, 1980). Rituals induce “acceptance, compliance, or at least forbearance with regard to any overt challenge” (Bell, 1997), as can be seen right from the beginning of the pandemic. A convincing example from Pakistan is the first media briefing from the Prime Minister (PM) of Pakistan in March 2020, in which he repeatedly stated, *Ghabrana nahi hai* (don't panic,) or “there is no reason to worry” (Dawn, 2020b). Thereafter, the PM and health officials repeatedly conducted meetings, introduced and implemented various preventive measures, and used *soft* and *hard* power to get people to follow them. On the one hand, the public was *asked* to behave in a particular way (soft power), and on the other hand, it was strongly *declared* that there would be legal consequences against those who failed to comply (hard power) (see Bates, 2020), as the government even deployed the army and the police to ensure compliance. The standard operating procedures (SOPs) that the Pakistani government created, namely repetitive hand washing, mask wearing, physical distancing, social isolation, and how to deal with a dead viral body, can all be seen as rituals of containment, as they cause people who follow them to behave in ritualized ways.

Digging deeper, we can see that these rituals of containment demonstrate what Turner (1974, p. 37) called “social drama,” which he defines as “units of harmonic or disharmonic social process, arising in conflict situations.” Turner divided a conflict into four stages: breach; crisis; redressive action; and either reintegration or schism. During such conflict situations, public action contains an arrangement of processual acts and scenes with dynamic shifts in scripts, characterizations, rhetoric, and symbolism.

The situation of COVID-19 in Pakistan, as elsewhere, reveals a constant negotiation of just such a social conflict. In terms of the four stages of a conflict proposed by Turner, the pandemic can be seen as the “breach” of normal, as it has affected the societal fabric, and brings “fundamental aspects of society, normally overlaid by the customs and habits of daily intercourse, into frightening prominence” (Turner, 1974). The “crisis” has appeared in the country as people and government become divided in this social conflict, as they take differing positions and dramatize them. Turner sees a liminal quality in these kinds of public crises, “betwixt and between” more or less stable social process phases. Within this liminality, social upheaval occurs as antagonists criticize each other, with each group trying to generate some sort of societal change, which is what Turner would term “redressive action.” In the resulting “schism,” the government has criticized the public for failing to comply and some citizens have criticized the government for trying to make them comply. For example, some people in Pakistan consider COVID-19 as a sort of national or global “plot,” while the government tries to show them that it is a reality (see Ali, 2020b,c, 2021). Both camps employ differing types of logic and “proof.” In such ideological clashes, one can see the social dramas of COVID-19 denying movements and COVID-19 accepting movements.

Analogous to the world, people in Pakistan have been continuously working and reworking with the tensions between “normals” and “abnormals” during the pandemic. At its beginning, there was great fear, and many people, especially in urban areas, were adjusting their acts according to the constantly realigning imaginaries. They created different strategies to make sense of an unfolding critical situation (Ali, 2020b,c). These processes and the types of social confusion they generated extensively continued during the second waves of the pandemic. Yet by now, although COVID-19 is still a major threat, and turning again to the four conflict stages as defined by Turner (1974), we can see that many people have “reintegrated” either into the “old normal” or “new normal” patterns of social life, depending on how seriously they take that threat. Yet, though reintegration has occurred for some, the conflict “schism” remains for others. In their process of reintegration, those who believe that COVID-19 is real have become accustomed to following the rituals of containment of the government, namely the SOPs listed above. Those who do not, who live mainly in rural areas, continue the schism by living life as usual, for example, continuing to arrange marriages and hold other large group gatherings. Many rural people in Pakistan do not believe that COVID-19 is real; such groups of course are at major risk of viral spread (Ali, 2020c).

As Liu (2020) has found in China, rituals of containment in Pakistan have demonstrated significantly contested public domains in that ongoing schism. Drawing on a repertoire of socio-cultural forms, e.g., traditional values, religion, geopolitics, history, and current affairs at the national and global levels, the major players negotiating the pandemic include the Pakistani government, the opposition political parties, laypeople, and “netizens” who get their information from the Internet. The dealings of the Pakistani government or the “redressive actions” very much resemble the findings by Liu (2020) about the Chinese government, which, as a strong actor, has used its political hegemony to control or censor everything related to the pandemic *via* its own rituals of containment (though Liu does not use that term), including manipulating the epidemiological data about the numbers of infections and deaths (Ali, 2021), thereby containing information about the true extent of viral spread. Although netizens and laypeople have criticized the Pakistani government, especially its fabrication of facts, the government has given an outstanding performance of showing both laypeople and international agencies, *via* statistics demonstrating a very low (0.0025) infection rate and *via* the use of various metaphors and rituals of containment, that it has done a great job of dealing with the pandemic.

From a more global perspective, the performance of the Pakistani government can be seen as a “meta-theater,” which Turner views as a mode of reflexivity for, of, and by everyday actors to communicate consciously to give spectators active roles and make them part of the performance. This meta-theater, which involves dialectics of trust and mistrust, criticism, and appreciations, may be over sooner or later, but it is always recorded in what I have called *societal memory* (Ali, 2020a): the good or bad characters, scenes, and acting are preserved in this memory and reappear during similar situations that re-evoke them. Social dramas involving what can be called either *micro-* or *meta-performances* can pause for a time, then later re-appear in different forms when events reawaken that societal memory, as COVID-19 has done in Pakistan by reawakening societal memories of, for example, the highly contested vaccination campaigns that have been repeatedly carried out throughout the country. These vaccination campaigns, which I have intensively studied, can themselves be seen as rituals of containment, in this case, efforts to contain the spread of infectious diseases such as measles, polio, mumps, and rubella. They became major sites of conflict and social drama after the fake vaccination campaign conducted by the US CIA to discover the whereabouts of Osama-bin-Laden, and for many other reasons too complex to discuss here (but see Ali, 2020a,c). A major and tragic part of this social drama was the killing of around 100 vaccinators and their security guards. This schism around vaccination is reflected in the schism around COVID-19 and will again become a major issue once COVID-19 vaccines are available. Many people, especially in the rural areas of Pakistan, are likely to refuse these vaccines and their rituals of containment, thereby generating yet another major social drama around COVID-19 and its “political pandemic”.

## Background and Context, Results, and Discussion

Reporting its first COVID-19 infection in February 2020, by January 2021, Pakistan had reported COVID-19 infection in over 543,000 people, out of whom around 11,600 have “officially” (according to questionable government statistics) died (Johns Hopkins University, 2021). Like many governments, the Pakistani government has performed various rituals of containment: these have included banning travel; the implementation of lockdown; the deployment of military and police to enforce lockdown; a later switch to “smart lockdown” (called “targeted lockdown” in the US); the creation of a Corona Relief Tiger Force; and educating people about the severe consequences of violating Article 188 of the Pakistan Penal Code, which demands that people follow the government given SOPs, violations of which incur a penalty of 6 months in prison, a fine, or both.

Many people who violated the SOPs have been booked under this Article. For instance, over 100 laypeople were arrested in Sindh for violating lockdown measures and were irrationally crammed into a police vehicle without masks; the police said that they did not have a sufficient number of masks (The News, 2020). Similarly, in another major social drama in October 2020, the government arrested around 400 workers of opposition parties who had planned a grand rally in Punjab (Tahir, 2020).

Government rituals of containment have also included market closures; banning group gatherings; distributing food items among daily wage workers; the approval of the PKR financial assistance program of Rs. 1.2 billion (Ali, 2020a; Ali and Davis-Floyd, 2020); and monitoring the burial of dead viral bodies, which this article addresses. During the pandemic, many tensions and contestations have arisen between federal and regional governments, and between citizens and these governments, along with negotiations between the local and global worlds. Various local narratives have emerged regarding COVID-19 being considered a “supernatural test,” a “Western conspiracy,” a “Jewish plot,” or a “political game” of the Pakistani government to gain more foreign aid (Ali, 2020b,c). Due to these interpretations, a substantial number of people do not follow SOPs, such as not wearing a mask or maintaining physical distancing. In March 2021, I observed in a famous chain of grocery shops where out of twenty people, including sellers and buyers, only one person was properly wearing a mask, while the rest were without a mask or wearing it at their chin. When I asked the person in charge of that shop, he replied, “Boys are coming here and there. It is difficult for them to wear a mask.” He himself was standing at the cash counter, and his response about him not wearing a mask was related to the religious explanation: “I am about to leave for the *Juma* (Friday) prayer [that was still almost 2.5 h from this encounter]. Allah is great. If you offer *Namaz* (prayers) and *Dua* (supplication), then COVID-19 does not affect you”.

Likewise, in March 2021, I have been taking private cabs run by *Kareem* in which out of almost 40 drives, only two drivers wore a mask without my asking. Two of them even had no mask to wear, thus, I provided them. One driver, who seemed to be in his forties, with a long black beard also stated while smiling when I asked him to wear a mask, “Sir! Where is COVID-19? These are just rumors. If there is then it can be controlled *via* performing prayers. I have been observing prayers; hence, I haven't contracted the virus during the entire year. Even no one from my family has been sick.” Within the political pandemic in Pakistan, these reflect different levels and scales of politics and mistrust and their ensuing social dramas.

### Social Drama: Invoking Religion and Patriotism

The social drama around COVID-19 and the political pandemic in Pakistan started as soon as the outbreak began in the country. The government immediately took to the media. Among the first containment rituals were the media briefing from the PM (as mentioned earlier) and a nationally televised broadcast of a *Namaz Aaft* (a prayer to reverse trouble or a curse) at the PM House of Pakistan^1^. This ritualistic prayer can be seen as the “governmental etiology” of COVID-19 that the pandemic is a “supernatural curse,” or “test.” Foster (1976, p. 773) might have called it a “personalistic etiology” since it shows the “belief that all misfortune, disease included, is explained in the same way” in which “illness, religion, and magic are inseparable.” I think one must replace “magic” with “magical politics” to add to these forms of etiology. This prayer can be ethically viewed as a magical spell cast both as an attempt to end the pandemic and to turn the attention of the laypeople toward prayer to bear any loss; in other words, “don't blame the government for failing to contain the virus, but rather believe that it is a ‘supernatural test or punishment’ and therefore the will of Allah.” Similar findings were observed by Malik (2020) that many people in Pakistan perceived the pandemic as God's wrath and continued religious practices, even believing that those who die due to COVID-19 during these worships would be rewarded by God.

In addition to invoking religion, the government has also invoked patriotism by continually using militarized terms such as “soldiers” fighting a “war” (Ali and Davis-Floyd, 2020). During that first media briefing in March 2020, the PM emphasized, “I want to tell all of you, this virus will spread… We have to win this war as a nation” (Dawn, 2020b). The virus is an “enemy” that needs an “army” to fight against it, hence the creation of the Corona Relief Tiger Force. Using war metaphors certainly is not specific to Pakistan, as almost every government worldwide has used them. These war metaphors instantiate the pandemic as a major social drama, and substantially (re-)frame our thoughts and actions in both positive and adverse ways. They can increase perceptions of COVID-19 as a serious and urgent “threat,” or they may lead to a fatalistic attitude, causing people to avoid implementing any preventive measures but rather to rely on the will of Allah (Semino, 2021).

Regarding the rituals of containment of the government for dealing with dead viral bodies, they are clearly based on science (Vidua et al., 2020). The government produced a three-page document, *Burial and Safe Management of [the] COVID-19 Dead Body*, containing guidelines about “the preparation of the [dead] body,” “Autopsy, including engineering and environmental controls,” and “Burial” (Government of Pakistan, 2020, p. 1). Yet, this science has been applied in discriminatory ways regarding who should or should not be given the “dead viral body.” When the COVID-19 outbreak was still escalating in Pakistan, various rumors spread that doctors were “injecting a drug” into the patients that caused them to die. During the “second wave” (Ali, 2020c), when more people were infected and admitted to hospitals, and some died, it was locally perceived that the government did not return the dead viral bodies to their *Waris* (heirs) to perform the final mourning rituals, unless they were wealthy and powerful elites. In contrast, the socio-economically and politically poor have complained about not receiving the dead viral bodies of their beloved ones for a proper burial. One of my female interlocutors who survived COVID-19 shared, “The government was returning the dead bodies to rich people only, but not to the poor. This indeed is not justice.”

In some exceptional cases, one of which I illustrate later on, the government gave the family an empty coffin. This containment of dead viral bodies is an example of this invisible or structural violence and of the emotional/psychological coronavirus pandemic, especially when the family is tricked into believing that there actually is a body in the coffin they receive. Dealing with such bodies according to scientific evidence and guidelines is justifiable; lying to the families is not. Nor is the discriminatory practice of giving what I am terming *influential* dead viral bodies to their families, but not the dead viral bodies of the poor. That discrimination, which forms part of the “structural pandemic,” has been purely power-driven, as I show in the section below.

### “An Empty Coffin”: Who Owns the Body?

According to the government record, around 11,000 people had died due to COVID-19 in Pakistan as of January 2021. If the death rate were calculated according to the total population of the country, as previously noted, the result would be 0.000025%. These epidemiological data are “success stories” for a country of 220 million, where around 25% of its people live below the poverty line, the doctor–patient ratio is around 1/1,000, and the hospital bed per patient ratio is around 1/1,600 (Ali, 2020c). These figures related to COVID-19 indeed are a source to ponder, as they reveal the *data fabrication* performed by the government, which is designed to give an impression that the situation is under control in Pakistan as compared to other countries (Ali, n.d.).

In a previous article, I explained the “symbolic ownership” of the government in the case of a gentle lady who died due to COVID-19 and her family members did receive her viral dead body, with specific instructions to follow SOPs (Ali, 2021). Yet, as previously noted, as part of the structural pandemic, not all people have received the dead bodies of their beloved family members. Some dead viral bodies have been buried by government-chosen teams without the presence of family members.

During my data collection, I came across an account of a family who had lost their young son. Developing critical symptoms, he was admitted to a government hospital in Punjab province. His situation deteriorated over time, and ultimately, he succumbed to death from COVID-19. The *Waris* (heirs) were informed that their family members had died. With heavy hearts, some of them went to the hospital to collect the dead body. However, painfully, they were refused. As a result, the family staged a protest, and the government was compelled to hand over the dead viral body (again, by “dead viral body,” I mean a body that has died from COVID-19 and is still infectious) to family members on the condition that the body be packed in a coffin by the government. This reasonable condition stemmed from the fact that dead viral bodies, if exposed for viewing, might constitute a significant risk factor for those performing burial rituals, who might succumb to the desire to touch or kiss the body of their loved one, as used to be commonly done pre-COVID.

Considering it as a kind act of the government, the family agreed. The coffin was delivered, but it contained no glass window to view the face. It is necessary to mention that in Pakistan, viewing the face of the dead body is a socio-culturally valued practice and a source of religious rewards (Ali, 2021). And a coffin usually has a small piece of glass in it for this purpose. Thus, having no glass window was a puzzle for the family. Everyone accepted the order of not viewing the face placed by the government, except the sister of the deceased person. My interlocutor stated:

She [his sister] lived in a different city, and she had not seen her brother for many months. Hence, she emphasized seeing her brother's face. She continually cried and hugged the coffin. Since no one obliged to help her to open the coffin, she did this job on her own. And a most surprising thing occurred when the box was open. [My interlocutor sighs.] The coffin was empty. This was a great shock for the family. Consequently, they protested again, but nothing was given back to them. This is the situation that led me not to visit a hospital although I was infected and do have comorbidities such as cardiovascular problems, diabetes, and cancer. I remained at home. I wanted to die at home. During this time, government officials frequently visited my house many times, but I showed that I am okay.

In May 2020, the Pakistani media broadcast similar news about another such social drama, in which a mob attacked the Jinnah Postgraduate Medical Centre (JPMC) in Karachi after they were not given the dead body of their beloved family member who died due to COVID-19 (Dawn, 2020a). The story mentions the views of one doctor from that hospital, who said, “When a COVID-19 patient dies, the hospital administration calls the district health officer, who arranges the *ghusl* (bathing of a body) and burial with Edhi staff. They bury the body in a far-off graveyard” (quoted in Dawn, 2020a).

Constituting parts of the emotional/psychological and social pandemics, this fear of not receiving the dead bodies of their COVID-19-infected family members led many people not to disclose the deaths of those loved ones who died at home due to COVID-19. One interlocutor shared:

Although one of our acquaintances died due to COVID-19 at home, their family members hid the cause. Interestingly, most family members, acquaintances, and friends knew that the person died due to COVID-19. Despite that, those who could attend the funeral rites came. It appeared that the family was scared by the government that it may take the body and bury it on its own.

During my data collection, a substantial number of people shared such accounts of hiding COVID-19 infection and information about those family members who died due to this virus. This hiding can be seen as local level censorship to avoid governmental control of the dead viral body and as the ritual of containment of the family; in this case, the “containment” is not to prevent viral spread, but rather to contain the news of the cause of death.

## Conclusion

In this article, I have shown that that COVID-19 is not one pandemic but many, namely medical, economic, social, structural, emotional/psychological, and political, and have demonstrated some of the ways in which all of these pandemics play out in Pakistan and the social dramas they entail. Borrowing from various eminent anthropologists and building on their critical concepts, such as ritual (Leach, 1966; Bloch, 1974; Geertz, 1980; Davis-Floyd, 2003 [1992]); social drama (Turner, 1974, 1982, 1985); and structural violence and social suffering (Kleinman et al., 1997; Farmer, 2004), I have developed a new concept that I call *rituals of containment*, in part to show how the Pakistani government has invoked both religion and science in its political responses to the pandemic, revealing discrimination and the manipulation of body politics (Scheper-Hughes and Lock, 1987). This is a good example of the regulation and control of individuals and populations, which Foucault (1990) calls “bio-power.”

I have noted how these rituals of containment include all of the SOPs developed by the government to contain the viral spread. I have presented the government as the protagonist of COVID-19, in which it has hegemonic power to censor and control the behaviors of the people, epidemiological data about COVID-19, and viral dead bodies, and have illustrated some of the social and political schisms around that control (see for example Jamil and Appiah-Adjei, 2020). I conclude that dealing with such bodies according to scientific evidence and guidelines is justifiable; the problem lies in the discriminatory and contradictory government policies and practices, which in my analysis are part of both the political and the structural pandemics in Pakistan. Although they often do, governments should avoid lies that has critical consequence and do not discriminate against their vulnerable and marginalized populations. I envision a world in which structural disparities and discrimination cease to exist, and in which all rituals of containment are both evidence-based and widely implemented by popular consent.

## Study Limitations

Although this study is a part of a major project on COVID-19, the results cannot be generalized. Nonetheless, these perspectives reveal considerable evidence of how COVID-19 has been dealt with in Pakistan. Based on the provocations in this article, more anthropological and sociological studies can be carried out in Pakistan and other countries to explore the interplays among religion, science, and politics and how they are intertwined in social dramas and rituals of containment.

## Data Availability Statement

Since these data are part of my long project, they are not shareable. However, most of the data are reflected in the article. Requests to access the datasets should be directed to inayat_qau@ahoo.com.

## Ethics Statement

The studies involving human participants were reviewed and approved by The National Bioethics Committee of Pakistan. Written informed consent for participation was not required for this study in accordance with the national legislation and the institutional requirements.

## Author Contributions

IA designed the study, reviewed the literature, drafted, and revised the article.

## Conflict of Interest

The author declares that the research was conducted in the absence of any commercial or financial relationships that could be construed as a potential conflict of interest.

## Footnotes

^1^This house is in actuality an office for the PM, not his place of residence.
